# Ductile Lightweight Ti_x_(AlCrZrV)_100−x_ Medium Entropy Alloys with Superior Specific Yield Strength Through Compositional Tuning and Thermomechanical Treatment

**DOI:** 10.3390/ma19122644

**Published:** 2026-06-19

**Authors:** Po-Sung Chen, Ming-Che Li, Jason Shian-Ching Jang, I-Yu Tsao

**Affiliations:** 1 Institute of Materials Science and Engineering, National Central University, Taoyuan 320, Taiwan; 2 Department of Mechanical Engineering, National Central University, Taoyuan 320, Taiwan

**Keywords:** lightweight, medium-entropy alloys (MEAs), microalloying, thermomechanical treatment (TMT), mechanical properties

## Abstract

In this study, the Nb from the lightweight Ti_65_(AlCrNbV)_35_ medium-entropy alloy was replaced with Zr to create lower-density Ti*_x_*(AlCrZrV)_100−*x*_ (*x* = 65, 67, 70, or 75) alloys. All alloy ingots were fabricated through vacuum arc melting and drop casting. X-ray diffraction analysis revealed all as-cast alloys exhibited only a single body-centered cubic structure. As the Ti content increased, the strength of the as-cast alloys decreased from 1247 to 981 MPa, whereas their elongation marginally improved. Moreover, the mechanical properties of these alloys were considerably enhanced through thermomechanical treatment (50% hot rolling and 80% cold rolling) and then rapid annealing at 700 °C, 800 °C, or 900 °C. An increase in the annealing temperature led to a notable decrease in the yield strength of the alloys but a considerable increase in their ductility. Ti65, Ti67, and Ti70 alloys annealed at 700 °C or 800 °C exhibited a yield strength of ≥1200 MPa and a ductility of ≥10%. Of the fabricated alloys, the Ti67 alloy annealed at 700 °C exhibited the optimal mechanical properties (yield strength of 1552 MPa and ductility of 13.6%). It exhibited low density (4.89 g/cm^3^) and a specific yield strength of 317 MPa·cm^3^/g, thus demonstrating considerable potential for transportation and energy applications.

## 1. Introduction

The use of high-performance lightweight materials is essential for reducing costs and environmental pollution and improving efficiency in the transportation and energy industries [1,2]. However, traditional materials have limitations such as low strength or high weight, which restrict the design and development of vehicle or equipment [3]. Composite materials exhibit high performance and have been widely used in various industries; however, high costs, maintenance difficulties, and inherent constraints associated with their raw materials limit their applications [4,5]. Therefore, the fabrication of novel high-performance lightweight materials is a crucial concern in industry.

Most of the traditional alloys are composed of one major element and small quantities of several other elements. Until 2004, Yeh and Cantor et al. proposed the concept of high-entropy alloys (HEAs), also called the multi-principal element alloys (MPEAs) [6,7]. These HEAs or MPEAs consist of at least five principal elements, breaking through the conventional alloy design concept and significantly expanding its boundaries [8]. In addition, these alloys exhibit phase stability and excellent material properties, demonstrating high potential for further development [9,10,11]. Researchers have developed nonequiatomic HEAs and medium-entropy alloys (MEAs) on the basis of the original HEA concept. These alloys retain the characteristics of HEAs, broaden the scope of alloy design, and exhibit superior material properties to those of traditional alloys [12,13,14].

The mechanical properties of the alloys can be tailored and enhanced through several strengthening techniques to increase their applicability [15]. Solid-solution strengthening contributes a greater proportion of the yield strength of as-cast MEAs than does grain boundary strengthening because of the severe lattice distortion arising from atomic size differences [16]. The mechanical properties of the alloys can also be optimized through thermomechanical treatment (TMT) [17]. TMT involves hot working, cold working, and recrystallization processes. Hot working can eliminate casting defects and promote grain refinement, whereas cold working can accumulate the strain energy and provide the driving force for subsequent recrystallization annealing [18,19,20,21]. Furthermore, rapid annealing can be employed to refine recrystallized grains to thereby improve mechanical properties through grain boundary strengthening [22,23].

In the present study, the Nb from the quinary Ti_65_(AlCrNbV)_35_ MEA [24] was replaced with Zr to fabricate Ti*_x_*(AlCrZrV)_100−*x*_ (*x* = 65, 67, 70, or 75) alloys with lower density and a larger average atomic radius. Subsequently, the alloys were subjected to TMT to optimize the microstructure and mechanical properties. The purpose of this study was to explore lightweight MEAs exhibited low density (≤5 g/cm^3^), high specific yield strength (≥300 MPa·cm^3^/g), and high ductility (≥10%).

## 2. Experimental Procedure

### 2.1. Materials

A series of quinary Ti*_x_*(AlCrZrV)_100−*x*_ MEAs, designated as Ti65, Ti67, Ti70, and Ti75 (*x* = 65, 67, 70, and 75, respectively), were prepared using high-purity Ti (99.99%), Al (99.99%), Cr (99.99%), Zr (99.99%), and V (99.9%) as raw materials. The master alloys were produced through arc melting under a high-purity argon atmosphere by using a Ti getter to prevent oxidation. The arc melting furnace was designed and constructed in-house and powered by a Flextec^®^ 650X power supply (Lincoln Electric, Cleveland, OH, USA). These alloys were remelted six times to ensure compositional homogeneity. Subsequently, the ingots were drop-cast into a water-cooled copper mold under an argon atmosphere to produce plate ingots with the dimensions of 35 mm × 20 mm × 14 mm (length × width × thickness).

### 2.2. Processing

Before TMT processing, the MEAs were homogenized at 920 °C for 2 h under a high-vacuum atmosphere (<10^−5^ Torr) to eliminate elemental segregation, following which water quenching was conducted. Next, the thermal behavior was assessed using a differential scanning calorimeter (STA 449 F3 Jupiter, Netzsch, Bavaria, Germany) under a heating rate of 10 °C/s under an argon atmosphere. The homogenized ingots were then subjected to 50% hot rolling at 920 °C, followed by 80% cold rolling at room temperature (HR50CR80). Subsequently, the deformed samples were subjected to rapid annealing at 700 °C, 800 °C, or 900 °C (annealing time of 23, 32, or 63 s) with a heating rate of 15 °C/s and a high-vacuum atmosphere (<10^−5^ Torr), following which water quenching was conducted again.

### 2.3. Microstructure Characterization

The densities of the MEAs were measured on the basis of Archimedes’ principle. Their phase structures were then analyzed through X-ray diffraction (XRD) analysis (D2, Bruker, Billerica, MA, USA) by using Cu Kα radiation. The MEA samples were ground using silicon carbide (SiC) sandpaper with a grit size ranging from #80 to #1000. Next, each sample was polished using alumina (Al_2_O_3_) suspensions with particle sizes of 1, 0.3, and 0.05 μm in sequence. Subsequently, the microstructures of the samples were observed through optical microscopy (OM; BX51M, Olympus, Tokyo, Japan) and scanning electron microscopy (SEM; F50 Inspect, FEI, Hillsboro, OR, USA) with energy dispersive spectroscopy.

### 2.4. Mechanical Testing

The hardness of the MEAs was measured using a Vickers hardness tester (HV-115, Mitutoyo, Kawasaki, Japan) under a loading of 5 kg for 10 s. Their tensile mechanical properties were then tested using a universal testing machine (HT9102, Hung Ta, Taichung, Taiwan) under quasistatic loading with a strain rate of 1 × 10^−4^/s. The gauge dimensions of each specimen were 5 mm × 2 mm × 1.5 mm (length × width × thickness). For each process parameter, hardness measurements were performed 10 times, and the mean value and standard deviation were calculated. Compression tests were conducted 3 times for each condition.

## 3. Results and Discussion

The configuration entropy (${∆S}_{conf}$) of each fabricated alloy was calculated using the following equation:

(1) $$\Delta S_{conf}=-R\sum_{i=1}^{n}\left(c_{i}lnc_{i}\right)$$

where $c_{i}$ represents the atomic percentage of the *i*th element, and R is the ideal gas constant. The ${∆S}_{conf}$ values of the fabricated alloys ranged from 7.56 to 9.42 kJ·mol^−1^, decreasing marginally with an increase in the Ti content. With the exception of the Ti75 alloy (${∆S}_{conf}<$ 8.31), all alloys met the definition of an MEA [25]. The atomic size difference ($\delta_{r}$) for each sample was determined as follows:

(2) $$\delta_{r}=\sqrt{\sum_{i=1}^{n}c_{i}{\left(1-r_{i}/\bar{r}\right)}^{2}}$$

where $\bar{r}=\sum_{i=1}^{n}c_{i}r_{i}$, with $c_{i}$ and $r_{i}$ representing the atomic percentage and atomic radius of the *i*th element, respectively. The $\delta_{r}$ values of the alloys ranged from 4.49% to 5.28%, indicating the formation of a single solid-solution structure [26]. Table 1 presents the ${∆S}_{conf}$ and $\delta_{r}$ values of the fabricated alloys.

### 3.1. Characterization of the As-Cast Ti_x_(AlCrZrV)_100−x_ MEAs Before TMT

The densities of the fabricated alloys were measured on the basis of Archimedes’ principle, with the measured values being similar to the corresponding theoretical densities calculated using the mixing rule (Table 2). The fabricated alloys exhibited lower densities (≤5 g/cm^3^) than that of the Ti_65_(AlCrNbV)_35_ MEA (~5.1 g/cm^3^). This was attributable to the replacement of Nb with Zr, which has lower atomic weight.

**Figure 1:**
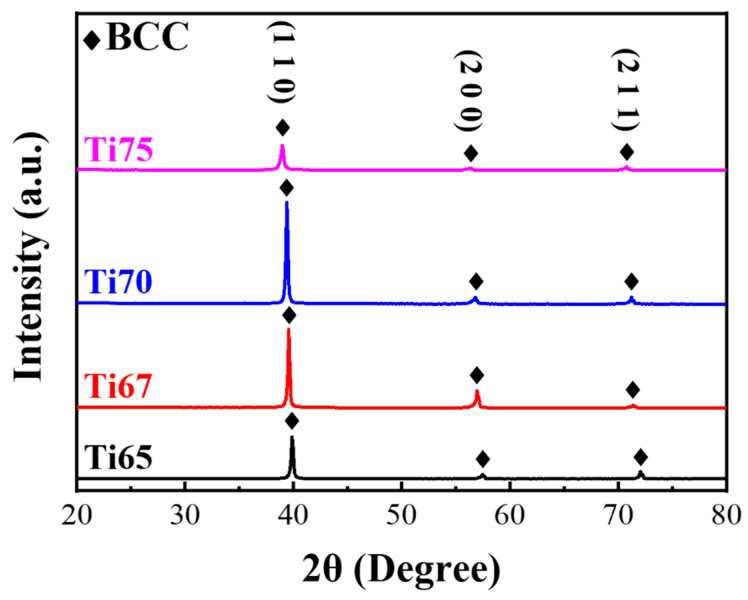
X-ray diffraction (XRD) patterns of as-cast Ti_x_(AlCrZrV)_100−x_ MEAs.

XRD analysis was conducted to examine the crystal structures of the fabricated alloys (Figure 1). The XRD patterns of all alloys contained the characteristic peaks of the body-centered cubic (BCC) structure. Furthermore, the (1 1 0) diffraction peak of the alloys gradually shifted toward lower diffraction angles (2*θ*) with an increase in Ti content. This displacement was attributable to Ti having a relatively larger atomic radius compared with the other elements, which led to Ti addition causing an increase in the lattice parameters of the alloys [27,28].

**Figure 2:**
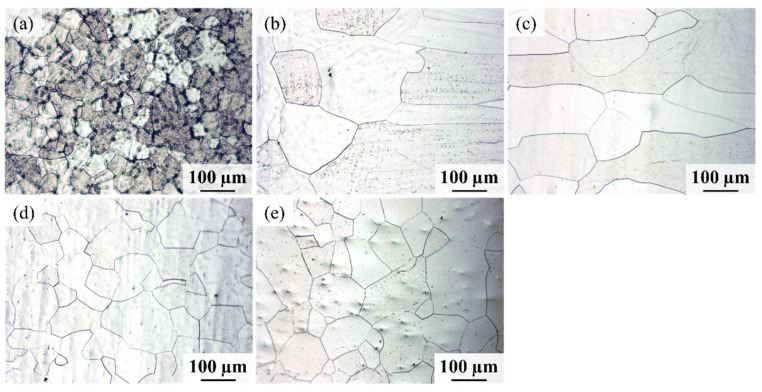
Optical microscopy (OM) images of Ti_x_(AlCrZrV)_100−x_ MEAs in the as-cast and homogenized (920 °C for 2 h) states: (**a**) as-cast Ti65 MEA (grain size: 47 ± 2 µm), (**b**) as-cast Ti67 MEA (grain size: 68 ± 4 µm), (**c**) as-cast Ti70 MEA (grain size: 87 ± 2 µm), (**d**) as-cast Ti75 MEA (grain size: 58 ± 5 µm), and (**e**) homogenized Ti65 MEA (grain size: 60 ± 6 µm).

**Figure 3:**
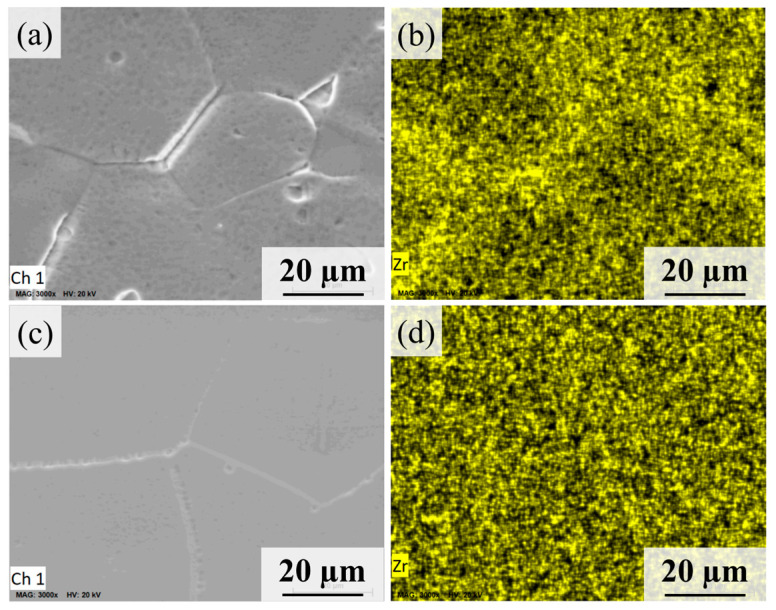
Scanning electron microscope (SEM) with energy dispersive spectroscopy (EDS) analysis of the Ti65 MEA: (**a**) as-cast/SEM, (**b**) as-cast/EDS, (**c**) homogenized/SEM, and (**d**) homogenized/EDS.

OM was employed to investigate the morphology and grain size of the fabricated alloys (Figure 2). The OM images revealed that no precipitates formed within the grains or along the grain boundaries; however, a dendritic structure was produced because of the insufficient cooling rate during solidification, which led to elemental segregation [29]. In addition, the grain sizes of the alloys were estimated using the linear intercept method, with the Ti65, Ti67, Ti70, and Ti75 alloys exhibiting grain sizes of 47 ± 2, 68 ± 4, 87 ± 2, and 58 ± 5 µm, respectively. No clear relationship was present between the grain size and Ti content of the fabricated alloys. Furthermore, the SEM with EDS was employed to further characterize the microstructure of the Ti65 alloy. The observations indicated obviously Zr segregation at the grain boundaries in the as-cast Ti65 alloy (Figure 3a,b).

**Figure 4:**
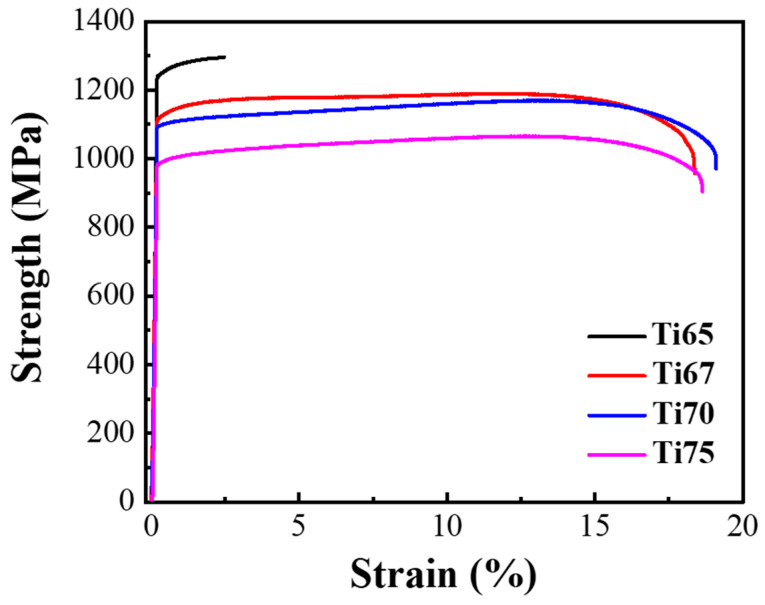
Tensile stress–strain curves of as-cast Ti_x_(AlCrZrV)_100−x_ MEAs.

**Figure 5:**
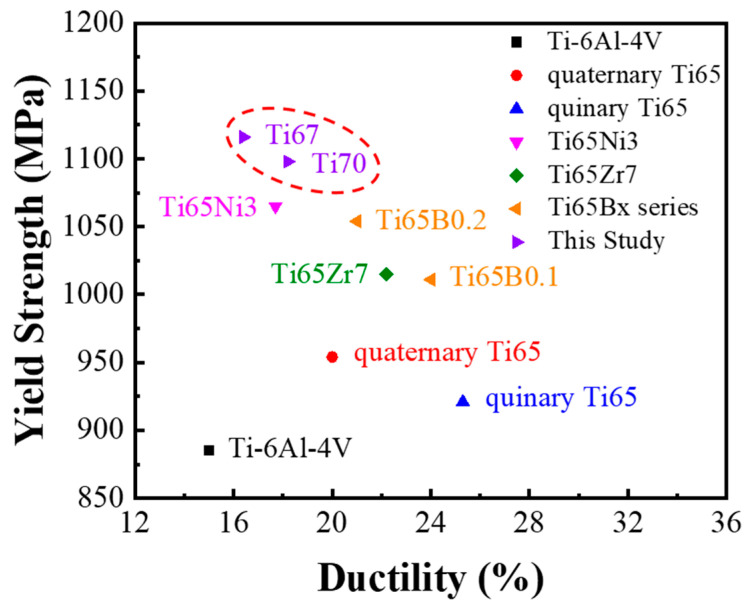
Yield strength and ductility of as-cast Ti_x_(AlCrZrV)_100−x_ MEAs, Ti-rich MEAs prepared in previous studies, and the commercial Ti–6Al–4V alloy [24,28,30,31,32].

Hardness and tensile tests were performed to evaluate the mechanical properties of the fabricated alloys (Figure 4 and Table 3). The replacement of Nb with Zr led to a considerable increase in hardness and yield strength (~18% and 35%, respectively); however, these parameters decreased with an increase in the Ti content, with hardness reducing from 374 to 320 HV and strength decreasing from 1247 to 981 MPa. Because Zr has a larger atomic radius than Nb does (155 pm vs. 145 pm), Zr induced more severe lattice distortion, which impeded dislocation movement and consequently enhanced the mechanical properties of the alloy. By contrast, Ti has a lower Young’s modulus than the other elements do; thus, Ti addition led to the weakening of the alloy. Moreover, the Ti65 alloy exhibited considerably lower ductility than did the other alloys. This result was attributable to the formation of excessive dendrites in the Ti65 alloy, which reduced its plasticity. Of the specimens, the as-cast Ti67 and Ti70 alloys exhibited the optimal mechanical properties; they maintained high ductility, and their yield strengths exceeded those of Ti-rich MEAs fabricated in previous studies and the commercial Ti–6Al–4V alloy (Figure 5) [24,28,30,31,32].

### 3.2. Characterization of the Ti_x_(AlCrZrV)_100−x_ MEAs After TMT

**Figure 6:**
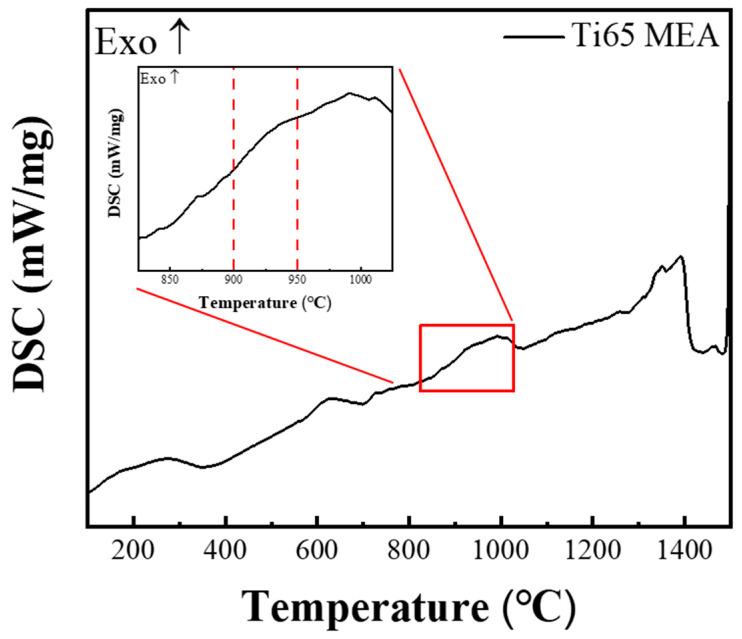
Differential scanning calorimetry curve of the as-cast Ti65 MEA.

The fabricated alloys were subjected to TMT to further improve their mechanical properties. Prior to this process, differential scanning calorimetry was employed to analyze the thermal behaviors of the alloys (Figure 6). The result revealed that no obvious thermal transition occurred between 900 °C and 950 °C. Therefore, the alloys were homogenized at 920 °C for 2 h to eliminate the dendritic structure before TMT. The OM image in Figure 2e and EDS analysis in Figure 3a,b indicates that the dendrites in the Ti65 specimen were effectively eliminated following homogenization, with the grain size increasing from 47 to 60 µm. After dendrite removal, the alloys were subjected to TMT. They were hot-rolled to achieve a 50% thickness reduction at 920 °C and then cold-rolled to achieve an 80% thickness reduction at room temperature. Subsequently, the alloys were subjected to rapid annealing at 700, 800, or 900 °C (annealing time of 23, 32, or 63 s, respectively).

**Figure 7:**
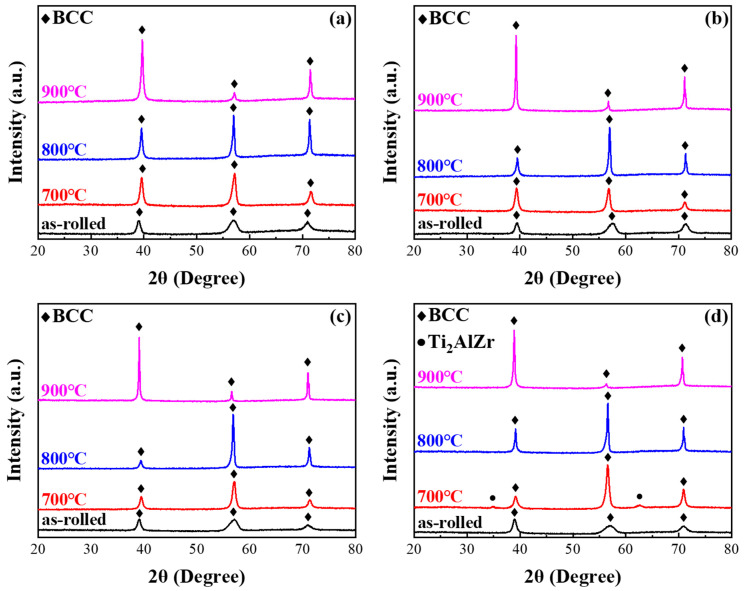
XRD patterns of Ti*_x_*(AlCrZrV)_100−*x*_ MEAs after their thermomechanical treatment (TMT): (**a**) Ti65, (**b**) Ti67, (**c**) Ti70, and (**d**) Ti75.

The XRD results indicate that all alloys maintained a single BCC phase after rolling process (Figure 7). The intensities and widths of the diffraction peaks decreased and increased, respectively, after this process because cold rolling introduced numerous dislocations (high dislocation density), which caused lattice distortion and, consequently, crystallinity reduction [33]. Annealing relieved the residual stresses and reduced the dislocation density in the alloys and then caused the nucleation and growth of new recrystallized grains. Therefore, as annealing temperature increased, the intensities and widths of the diffraction peaks gradually increased and decreased, respectively. A weak diffraction peak corresponding to Ti_2_AlZr precipitates was detected in the Ti75 alloy annealed at 700 °C [34]. In addition, the main diffraction peaks corresponded to (2 0 0) plane, which was attributed to grain deformation induced by cold rolling, resulting in a preferred orientation. By contrast, as the alloys were annealed at 900 °C, the main diffraction peak corresponded to the (1 1 0) plane, consistent with the as-cast state, which was attributable to recrystallized grains replacing the deformed grains caused by cold rolling.

**Figure 8:**
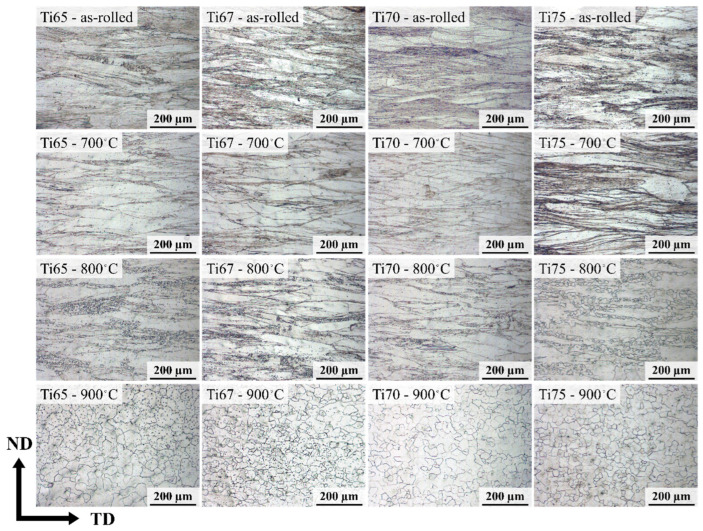
OM images of Ti*_x_*(AlCrZrV)_100−*x*_ MEAs after their TMT. As-rolled condition: hot rolled 50% and then cold rolled 80%.

The OM images revealed that the fraction of recrystallized grains increased with an increase in the annealing temperature (Figure 8). Cold rolling introduced a normal force in the normal direction (ND), which caused the grains in the alloys to exhibit slight elongation in the transverse direction (TD). After annealing at 700 °C, the alloys still contained excessive residual deformed grains, indicating this temperature was insufficient for inducing recrystallization. As the annealing temperature was increased to 800 °C, recrystallized grains formed on the boundaries of the residual deformed grains. The Ti75 alloy exhibited obvious recrystallization when it was annealed at 800 °C, which was attributable to its lower configuration entropy value compared with those of the other alloys. Because the configuration entropy of this alloy was less than 8.31 kJ·mol^−1^, it was not classified as an MEA and did not exhibit the sluggish diffusion effect [11]. After annealing at 900 °C, all alloys exhibited complete recrystallization, with all residual deformed grains replaced by recrystallized grains because of the sufficient energy in the annealing process.

**Figure 9:**
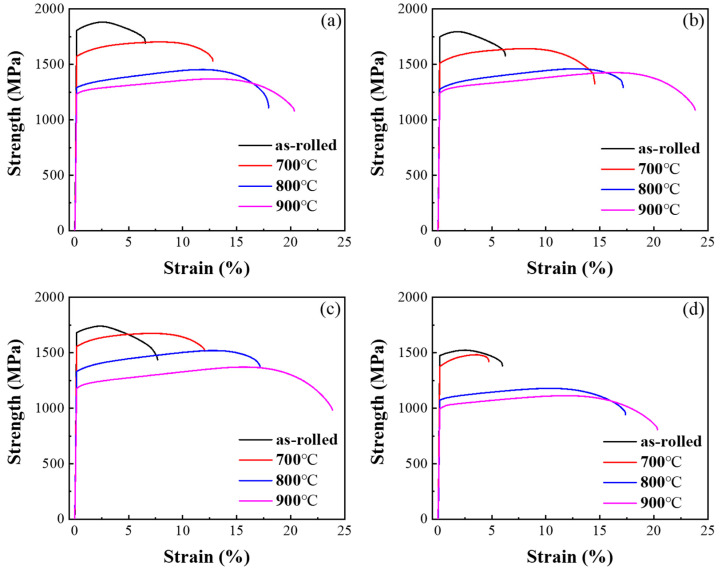
Tensile stress–strain curves of Ti_x_(AlCrZrV)_100−x_ MEAs after their TMT: (**a**) Ti65, (**b**) Ti67, (**c**) Ti70, and (**d**) Ti75.

The results of tensile tests conducted on the alloys subjected to TMT are depicted in Figure 9 and presented in Table 4. After cold rolling, the yield strengths of the Ti65, Ti67, and Ti70 alloys increased by approximately 600 MPa, whereas that of the Ti75 alloy increased by approximately 370 MPa. However, the ductility of all treated alloys except for the treated Ti65 alloy decreased to approximately 5%, suggesting that they became more brittle after cold rolling. In contrast to the other treated alloys, the treated Ti65 alloy exhibited an increase in ductility from 1.9% to 5.6% following the rolling process, indicating that elemental segregation caused substantial deterioration in the mechanical properties of the as-cast Ti65 alloy. When the Ti65, Ti67, and Ti70 alloys were annealed at 700 °C, their yield strengths decreased to approximately 1550 MPa, whereas their ductility increased to approximately 6%. The high temperature associated with annealing promoted recovery, resulting in a decrease in crystalline defects and the annihilation of dislocations, which reduced strength while increasing ductility [35]. However, compared with the ductility of the Ti75 alloy in the as-rolled state, its ductility decreased following annealing at 700 °C, which was attributable to the influence of Ti_2_AlZr precipitates. After the Ti65, Ti67, and Ti70 alloys were annealed at 800 °C, their yield strengths decreased to approximately 1300 MPa, whereas their ductility increased to approximately 17%. These results were attributable to recrystallization, as indicated by the OM observations (Figure 8). The residual deformed grains retained higher strength because of work hardening, whereas the recrystallized grains could accumulate more dislocations before rupture. The increased fraction of recrystallized grains enhanced the strain-hardening capacity of the alloys during deformation, delaying plastic instability and thus improving their ductility. Finally, when the Ti65, Ti67, and Ti70 alloys were annealed at 900 °C, their yield strengths decreased to approximately 1200 MPa, whereas their ductility increased to greater than 20%. These results were attributable to the fully recrystallized microstructure of the annealed alloys. Overall, the fraction of recrystallized grains increased with increasing annealing temperature, accompanied by a gradual reduction in strength and an improvement in ductility.

**Figure 10:**
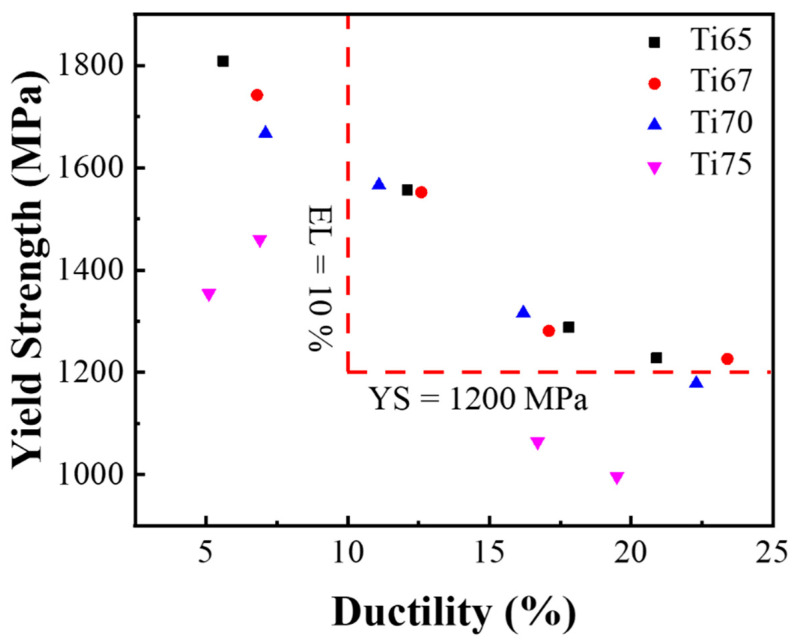
Yield strength and ductility of T_ix_(AlCrZrV)_100−x_ MEAs after their TMT.

**Figure 11:**
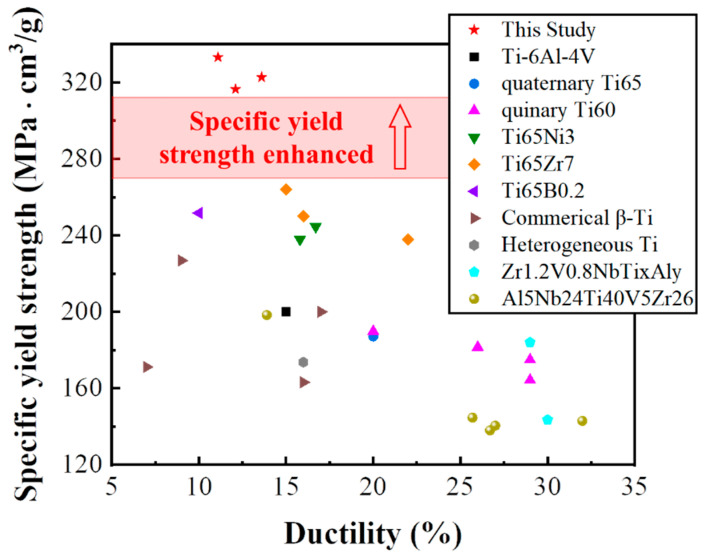
Specific yield strength and ductility of Ti_x_(AlCrZrV)_100−x_ MEAs, commercial Ti alloys, and other lightweight high-entropy alloys and MEAs [24,28,30,31,32,36,37,38,39].

In summary, the mechanical properties of the fabricated alloys were effectively improved following TMT (Figure 10). The alloys subjected to TMT exhibited excellent mechanical properties, demonstrating both high yield strength (~1200 MPa) and high ductility (~10%). Furthermore, because of their low density (≤5 g/cm^3^), the specific yield strength of the alloys could reach up to 300 MPa·cm^3^/g, exceeding those of commercial Ti alloys and other lightweight HEAs and MEAs [24,28,30,31,32,36,37,38,39] (Figure 11). Of the investigated alloys, the Ti67 alloy subjected to TMT followed by rapid annealing at 700 °C exhibited the optimal mechanical properties. It had an excellent specific yield strength of 317 MPa·cm^3^/g (yield strength of 1552 MPa) and a ductility of 13.6%, thus demonstrating considerable potential for energy and transportation applications.

## 4. Conclusions

In this study, a series of Ti*_x_*(AlCrZrV)_100−*x*_ MEAs (*x* = 65, 67, 70, or 75) were produced, and their microstructures and mechanical properties were investigated in the as-cast state, after TMT, and after annealing (following TMT). The conclusions of this study are summarized in the following:

1. The densities of the fabricated alloys were less than 5 g/cm^3^, indicating they were lightweight. The density decreased with an increase in the Ti content.
2. XRD results indicated that all fabricated alloys except for the Ti75 alloy exhibited a single BCC phase in the as-cast state and after TMT. The Ti75 alloy also contained a single BCC phase in the as-cast state; however, after TMT and thermal annealing at 700 °C, it also contained Ti_2_AlZr precipitates.
3. At as-cast state, the yield strength of alloys decreased with increasing Ti content; however, the Ti65 alloy exhibited poor ductility because of dendrite formation.
4. After TMT processing, the fraction of recrystallized grains increased with an increase in the annealing temperature. Meanwhile, the Ti75 alloy exhibited considerable recrystallization because of its low configuration entropy.
5. After TMT, the alloys exhibited outstanding combinations of yield strength (≥1200 MPa) and ductility (≥10%). Of the fabricated alloys, the Ti67 alloy subjected to TMT followed by rapid annealing at 700 °C exhibited the optimal mechanical properties. It had a specific yield strength of 322 MPa·cm^3^/g and a ductility of 13.6%, which highlights its considerable potential for transportation and energy applications.

## Figures and Tables

**Table 1:** Configuration entropy (${∆S}_{conf}$) and atomic size difference ($\delta_{r}$) values of Ti*_x_*(AlCrZrV)_100−*x*_ medium-entropy alloys (MEAs).

Composition	${∆S}_{conf}$ (kJ·mol^−1^)	$\delta_{r}$ (%)
Ti65	9.42	5.28
Ti67	9.08	5.13
Ti70	8.54	4.90
Ti75	7.56	4.49

**Table 2:** Densities of Ti_65_(AlCrNbV)_35_ and Ti*_x_*(AlCrZrV)_100−*x*_ MEAs [24].

Composition	Theoretical Density (g/cm^3^)	Measured Density (g/cm^3^)
Ti_65_(AlCrNbV)_35_	5.10	5.09 ± 0.19
Ti65	4.92	4.94 ± 0.04
Ti67	4.89	4.89 ± 0.09
Ti70	4.87	4.86 ± 0.04
Ti75	4.81	4.78 ± 0.06

**Table 3:** Mechanical properties of as-cast Ti_65_(AlCrNbV)_35_ and Ti*_x_*(AlCrZrV)_100−*x*_ MEAs [24].

Composition	Hardness (HV)	Yield Strength (MPa)	Ultimate Strength (MPa)	Ductility (%)
Ti_65_(AlCrNbV)_35_	317 ± 3	921 ± 11	1159 ± 14	25.3 ± 1.4
Ti65	374 ± 2	1247 ± 6	1289 ± 8	1.9 ± 0.7
Ti67	359 ± 2	1116 ± 10	1171 ± 13	16.4 ± 2.0
Ti70	346 ± 2	1098 ± 5	1180 ± 11	18.2 ± 0.9
Ti75	320 ± 2	981 ± 2	1059 ± 5	15.5 ± 2.3

**Table 4:** Mechanical properties of Ti*_x_*(AlCrZrV)_100−*x*_ MEAs after their TMT.

Composition	Processing	Yield Strength (MPa)	Ultimate Strength (MPa)	Ductility (%)
Ti65	as-rolled	1809 ± 2	1870 ± 11	5.6 ± 0.9
	700 °C	1557 ± 24	1668 ± 37	12.1 ± 0.8
	800 °C	1288 ± 2	1449 ± 4	17.8 ± 1.5
	900 °C	1228 ± 6	1372 ± 3	20.9 ± 0.6
Ti67	as-rolled	1742 ± 10	1786 ± 10	6.8 ± 0.6
	700 °C	1552 ± 39	1682 ± 40	13.6 ± 1.0
	800 °C	1281 ± 5	1459 ± 3	17.1 ± 0.1
	900 °C	1226 ± 34	1371 ± 45	23.4 ± 1.3
Ti70	as-rolled	1667 ± 14	1718 ± 22	7.1 ± 0.8
	700 °C	1566 ± 10	1676 ± 1	11.1 ± 0.9
	800 °C	1316 ± 14	1491 ± 30	16.2 ± 0.9
	900 °C	1178 ± 3	1354 ± 11	22.3 ± 1.4
Ti75	as-rolled	1460 ± 15	1503 ± 20	6.9 ± 1.0
	700 °C	1354 ± 22	1464 ± 22	5.1 ± 0.4
	800 °C	1064 ± 10	1166 ± 14	16.7 ± 0.7
	900 °C	996 ± 3	1105 ± 9	19.5 ± 0.8

## Data Availability

The original contributions presented in this study are included in the article. Further inquiries can be directed to the corresponding author.
